# Alkannin, HSP70 Inducer, Protects against UVB-Induced Apoptosis in Human Keratinocytes

**DOI:** 10.1371/journal.pone.0047903

**Published:** 2012-10-22

**Authors:** Yoko Yoshihisa, Mariame Ali Hassan, Yukihiro Furusawa, Yoshiaki Tabuchi, Takashi Kondo, Tadamichi Shimizu

**Affiliations:** 1 Department of Dermatology, Life Science Research Center, Graduate School of Medicine and Pharmaceutical Sciences, University of Toyama, Toyama, Japan; 2 Department of Radiological Sciences, Life Science Research Center, Graduate School of Medicine and Pharmaceutical Sciences, University of Toyama, Toyama, Japan; 3 Division of Molecular Genetics Research, Life Science Research Center, Graduate School of Medicine and Pharmaceutical Sciences, University of Toyama, Toyama, Japan; 4 Department of Pharmaceutics and Industrial Pharmacy, Faculty of Pharmacy, Cairo University, Cairo, Egypt; University of Tennessee, United States of America

## Abstract

Alkannin is an active constituent from the root extract of *Alkanna tinctoria* of the Boraginaceae family and it may have utility as a heat shock protein 70 (HSP70) inducer in living organisms. Here, the effects of alkannin-induced HSP70 on ultraviolet (UV) B (40 mJ/cm^2^)-induced apoptosis were investigated in human keratinocyte HaCaT cells. Pretreatment of cells with alkannin (1 µM) caused significant inhibition of UVB-induced apoptosis and caspase-3 cleavage. On the other hand, the addition of KNK437 (HSP70 inhibitor) reversed the action of alkannin increasing UVB-induced apoptosis in a dose-dependent manner. In addition, differences in gene expression associated with the suppression of UVB-induced apoptosis in the presence of alkannin were investigated using Gene Chip assay. Our results indicate that alkannin suppresses UVB-induced apoptosis through the induction of HSP70 in human keratinocytes, and therefore, we suggest the usefulness of using alkannin as an antiaging agent.

## Introduction

Exposure to ultraviolet (UV) light is a part of daily life, and under the steady decrease in ozone volume in the earth’s atmosphere and the consequent increase in the infiltrating harmful UV radiation, such research is essentially brought to the focus. The study of innate cellular protective responses and searching for alternative benign stimuli to trigger these responses against environmental hazards became an important branch of research in preventive medicine and for health promotion. UVB (280–320 nm) plays the central role in photo-damage including clinical sunburns, hyperpigmentation, erythema, plaque-like thickening, loss of skin tone, deep furrowing, and fine wrinkle formation, all of which constitute both clinical and cosmetic problems.

Keratinocytes, respond to elevated temperatures and other types of stress by synthesizing protective proteins known as heat-shock proteins (HSPs) [1]–[4]. HSPs are molecular chaperones that protect cells from extreme physiological, pathological, and environmental insults [5]. They have been reported to be of critical importance in the survival defense mechanisms of hepatocytes [6], cardiac myocytes [7], neurons [8], internal epithelial cells [8], [9], lung fibroblasts, and skin melanocytes and fibroblasts [10]. HSPs are involved in changing protein conformation, promoting multiprotein complex assembly and disassembly, inducing proteosomic pathways, translocating proteins, and guiding proper folding of nascent polypeptides [11]. These functions allow the cells to adapt to and survive during environmental changes [12]. Thus, HSPs play important physiological roles during both stress and aging [4], [13].

Alkannin is an active constituent isolated from the root extract of *Alkanna tinctoria*, family Boraginaceae. Boraginaceae species, including *Arnebia euchroma*, *Lithospermium erythrorhizon* and *Arnebia guttata*, are widely distributed plants in China. Alkannin has been used for centuries as a natural red dye and is used in Chinese popular folk medicine for its anti-inflammatory and antitumor activities [14]. Many studies showed that alkannin exerted antitumor effects by inhibiting cancer cell proliferation, inducing apoptosis, inhibiting DNA topoisomerase I/II activity, anti-telomerase activity and anti-angiogenesis [15]–[18]. Ahmed *et al*. has recently showed the results of screening 80 compounds of plant origin for their HSP70 inducing activity in human lymphoma U937 cells by Western blotting. Among these compounds, 5 compounds, namely; alkannin, oxymatrine, osthole, palmatine chloride, and shikonin, showed significant HSP70 upregulation [19].

In the present study, we examined the usefulness of alkannin in inducing HSP70 for the protection of keratinocytes against UVB-induced apoptosis. Moreover, we investigated the changes in gene expression in cells exposed to UVB light in the presence or absence of alkannin using a GeneChip for understanding the molecular mechanisms.

## Materials and Methods

### Reagents

Anti-HSP70 monoclonal antibody (code # SC-1060-R), anti-caspase-3 polyclonal antibody (code # SC-7148), and anti-β-actin monoclonal antibody (code # SC-130657) were obtained from Santa Cruz Biotechnology, Inc. (Santa Cruz, CA) Anti-NFκB mAb (code # 3033) and anti- IκB-α (code # 9242) were obtained from Cell Signaling Technology, Inc. (Beverly, MA); Geldanamycin was obtained from the Developmental Therapeutics Program, National Cancer Institute (Bethesda, MD, USA); Alkannin was obtained from Carl Roth GmbH and Co. (Karlsruhe, FRG); Oxymatrine was provided by Beijing SL Pharmaceutical Co. Ltd (Beijing, China); Osthole and palmatine chloride were purchased from Sigma Chemical Co. (St. Louis, MO); Shikonin was obtained from Ikeda Corp. (Tokyo, Japan); KNK437 (N-formyl-3,4-methylenedioxy-benzylidene-g-butyrolactam), an HSP70 inhibitor, was purchased from Kaneka Corp. (Osaka, Japan). All other reagents were of the highest commercially available purity.

**Figure 1:**
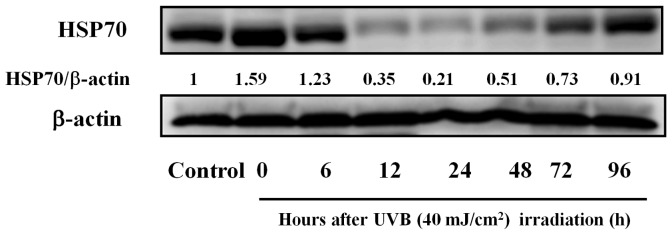
Induction of HSP70 protein by UVB radiation. Cells were exposed to UVB light (40 mJ/cm^2^) and harvested at various time intervals. Western blot analysis of HSP70 protein expression was then performed. Bands were quantified densitometrically and normalized to β-actin (n = 5).

### Cell Culture and Drug Treatments

HaCaT cells, an immortalized non-tumorigenic human keratinocytes cell line [20], were maintained in DMEM (Invitrogen, Carlsbad, CA) supplemented with 10% fetal bovine serum and 1% antibiotic mixture at standard cell culture conditions (37°C, 5% CO_2_ in a humidified incubator). One day before experiments, cells were collected and suspended in culture dishes in fresh medium at a concentration of 1×10^6^ cells/ml. In respective experiments, alkannin, oxymatrine, osthole, palmatine chloride or shikonin were added immediately after cell inoculation to allow for 24 h incubation. In inhibition experiments, KNK437 was added 6 h before UVB radiation in absence or in presence of drugs. All agents were dissolved in DMSO.

**Figure 2:**
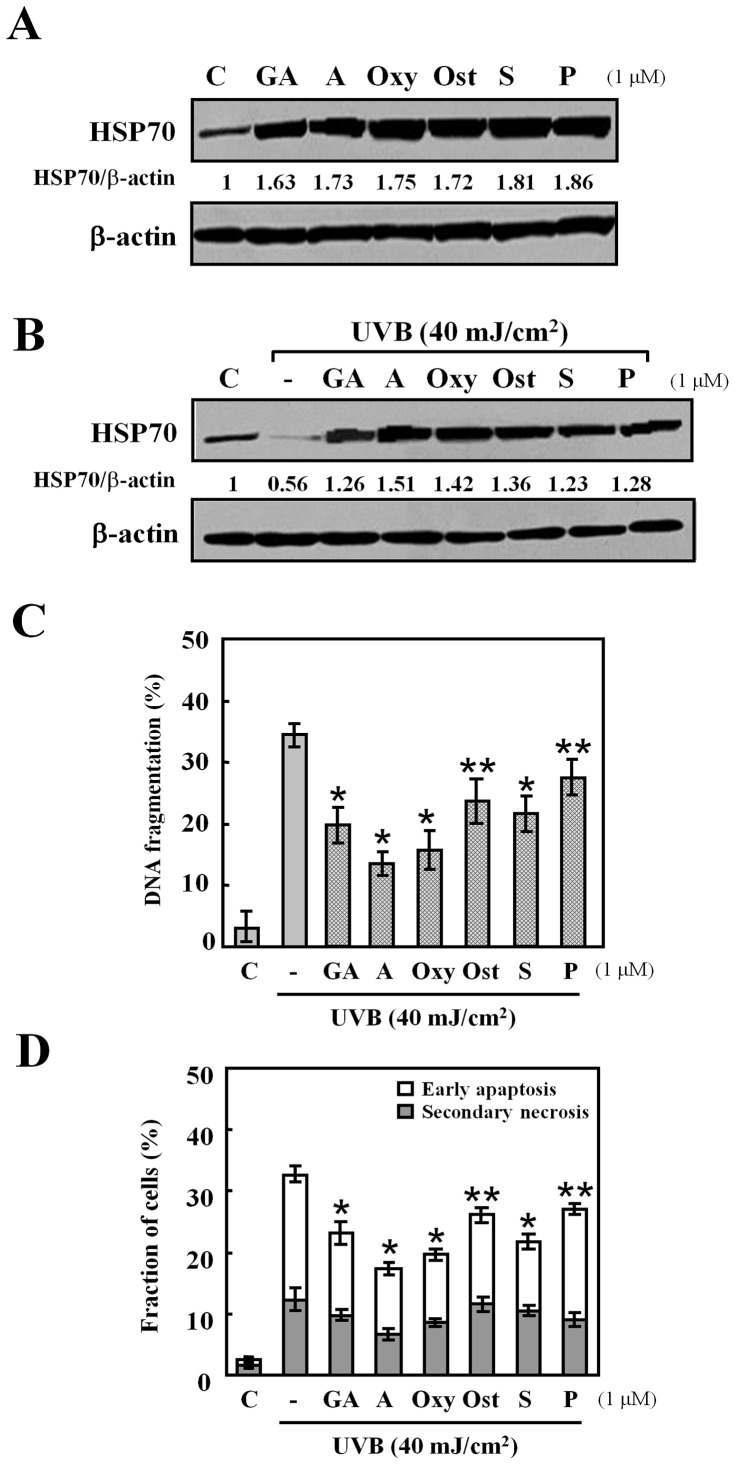
Effects of different HSP70-inducing agents. (A) Cells were treated with or without each HSP70-inducing agent: geldanamycin (GA), alkannin (A), oxymatrine (Oxy), osthole (Ost), shikonin (S) and palmatine chloride (P), at a dose of 1 µM for 24 h. Western blot analysis was performed with HSP70 protein (30 µg protein in each group). (B) Cells were exposed to UVB light (40 mJ/cm^2^) after the pretreatment with: geldanamycin (GA), alkannin (A), oxymatrine (Oxy), osthole (Ost), shikonin (S) and palmatine chloride (P), at a dose of 1 µM for 24 h. (C) DNA fragmentation assay was carried out after 6 h incubation at 37°C. Data are presented as mean ± S.D (n = 5). *p<0.001, **p<0.01. (D) Early apoptosis (cells positive for annexin V-FITC and negative for PI), and secondary necrosis (cells positive for both annexin V-FITC and PI). The percentages of early apoptosis and secondary necrosis were analyzed 6 h after UVB radiation by flow cytometry. Data are presented as mean ± S.D (n = 5). *p<0.001, **p<0.01.

### UVB Radiation

UVB radiation was carried out using a Spectrolinker XL-1,500 UV cross linker (Spectronics, USA), which emits most of its energy within the UVB range (280–320 nm) peaking at 312 nm. The UV dose was measured with a UVX Radiometer (UVP, Inc., Upland, CA). HaCaT cells were irradiated at a dose of 40 mJ/cm^2^ in phosphate buffer saline (PBS). After UVB radiation, cells were incubated in fresh medium in absence of drugs until analysis.

**Figure 3:**
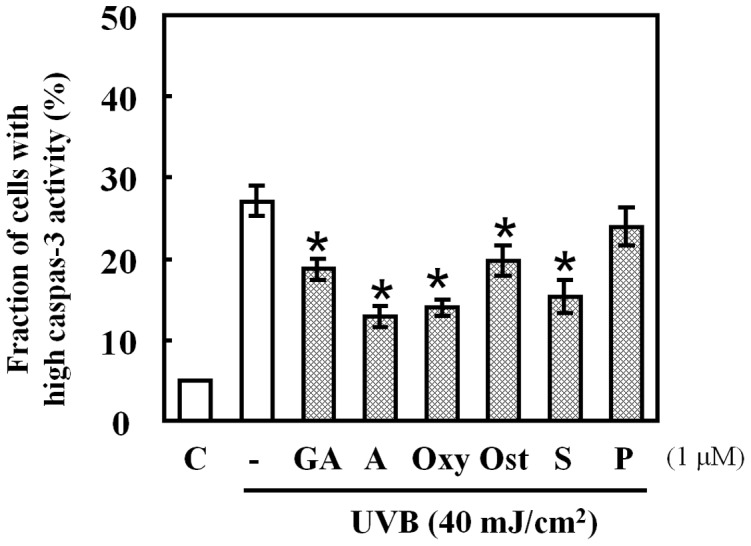
Effects of HSP70-inducing agents on caspase-3 activity in UVB-exposed cells. Cells pre-treated with HSP70-inducing agents at 1 µM for 24 h were exposed to UVB light (40 mJ/cm^2^). CaspGlowTM Fluorescein Active Caspase-3 Staining Kit was utilized to determine caspase-3 activity. Data are presented as mean ± S.D (n = 3). *p<0.001.

### DNA Fragmentation Assay

For the detection of apoptosis, the percentage of DNA fragmentation was assessed 6 h post treatment using the method of Sellins and Cohen with minor modifications [21]. In brief, approximately 3×10^6^ cells were lysed using 200 µl of lysis buffer and centrifuged at 13,000×*g* for 10 min. Subsequently, DNA from each sample in the supernatant and pellet was precipitated in 12.5% trichloroacetic acid at 4°C overnight and quantified using the diphenylamine reagent after hydrolysis in 5% TCA at 90°C for 20 min. The percentage of fragmented DNA for each sample was calculated as the amount of DNA in the supernatant divided by the total DNA for that sample (supernatant plus pellet).

**Figure 4:**
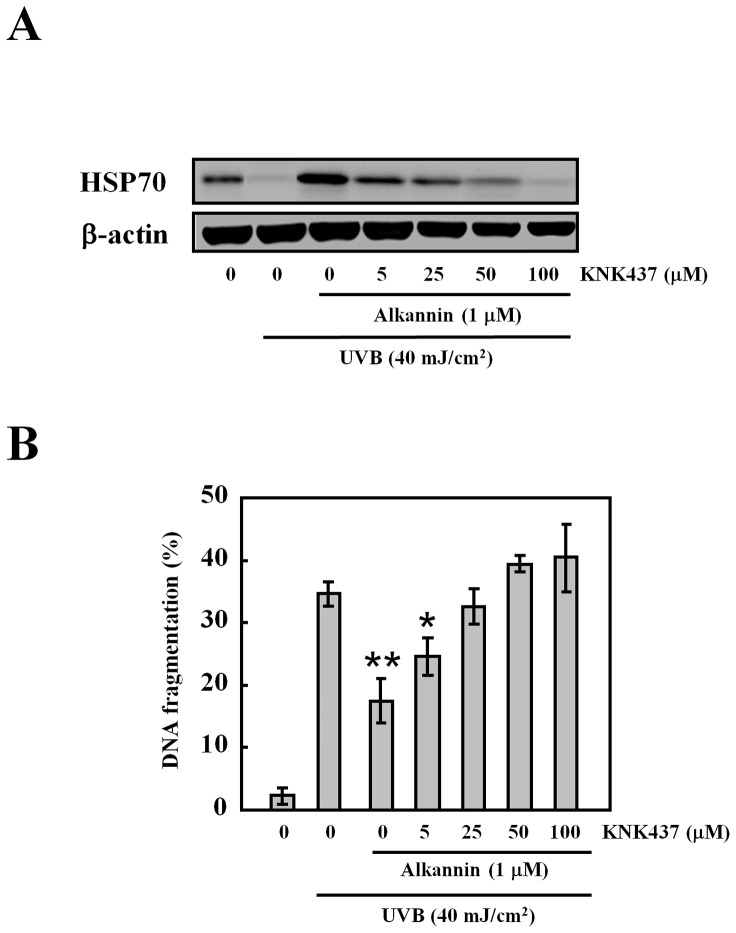
Effects of HSP70 inhibitor on apoptosis after UVB radiation. Cells were pretreated with KNK437 (HSP70 inhibitor) at different concentrations for 6 h and then exposed to UVB light (40 mJ/cm^2^). (A) Western blot analysis was performed 24 h after UV radiation with HSP70 protein (30 µg protein in each group). (B) DNA fragmentation assay of cells pretreated with both alkannin and KNK437 and finally exposed to UVB light (40 mJ/cm^2^). Data are presented as mean ± S.D (n = 5). *p<0.01, **p<0.001.

### Assessment of Early Apoptosis and Secondary Necrosis

To determine the percentage of early apoptosis and secondary necrosis, treated and control cells were collected 6 h post treatment and simultaneously stained with propidium iodide (PI) and fluorescein isothiocyanate (FITC)-labeled annexin V according to the instructions provided in the annexin V-FITC kit (Immunotech, Marseille, France). Finally, cells were analyzed by a flow cytometer (Beckman-Coulter EPICS XLTM) [22].

**Table 1:** Genes up- or down-regulated in HaCaT cells.

	Up-regulated		Down-regulated	
	Gene name	Fold	Gene name	Fold
Alkannin	BNIP3	10.83		
	SERPINE1	10.10		
	PDK1	4.33		
	PDK1a	4.11		
	BIRC3	3.33		
	BNIP3L	3.04		
UVB	SOD2	7.28	DNAJB12	0.33
	SERPINE1	6.87	DNAJB9	0.32
	GADD45B	4.59	HSPA8	0.26
	SOD2b	3.85	CDC25A	0.32
	SOD2b	3.85	CDC2	0.31
	DNAJC4	3.29	MDM2	0.30
	HSF1	3.10	BUB1	0.30
			CDC6	0.30
UVB and alkannin	HSPA13	6.95	MIR21	0.33
	RAB24	4.80	CENPE	0.33
	HSPA5	3.96	CENPA	0.32
	EPHA2	3.96	CCNB2	0.32
	CDKN1A	3.90	BNIP3L	0.30
	ATG5	3.36	PLK1	0.28
	XBP1	3.32	KIF20B	0.26
	BIRC5	3.26	CDC25C	0.24

The fold change is the ratio between control and each experiment area.

**Figure 5:**
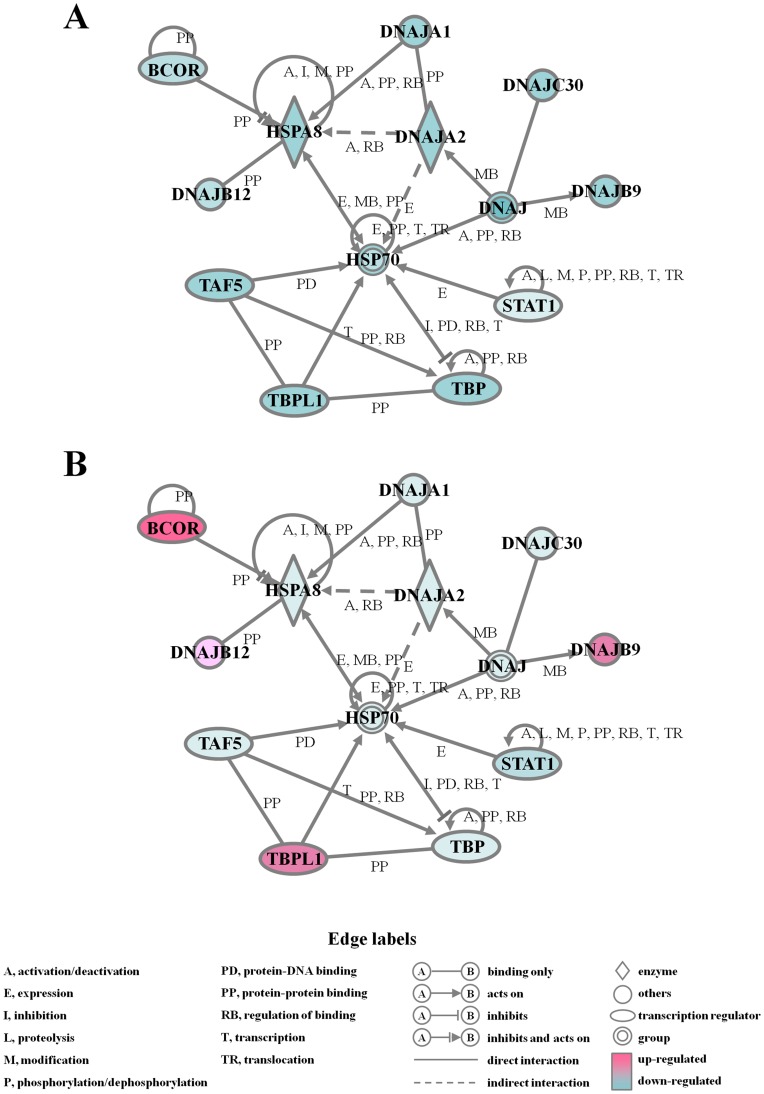
Changes in gene networks. Cells exposed to (A) UVB light (40 mJ/cm^2^), (B) alkannin pre-treatment and UVB radiation were analyzed by Ingenuity Pathways Analysis tools. The network is displayed graphically as nodes (genes or proteins) and edges (the biological relationships between the nodes). The node color indicates the expression level of the genes. Nodes and edges are displayed by various shapes and labels that represent the functional classes of genes and the nature of the relationship between nodes, respectively.

### Assessment of Intracellular Caspase-3 Activities

The CaspGlow™ Fluorescein Active Caspase-3 Staining Kit (MBL, Nagoya, Japan) was used to monitor the intracellular caspase-3 activity following the manufacturer’s recommendations. Briefly, pre-cultured cells were subjected to treatment, 300 µl of each of the samples and control cultures was aliquoted into a microtube after 24 h-incubation period, and 1 µl of FITC-DEVD-FMK was added into each tube followed by incubation for 30 min at 37°C in a 5% CO_2_ incubator. The samples were finally analyzed by flow cytometry [22].

**Figure 6:**
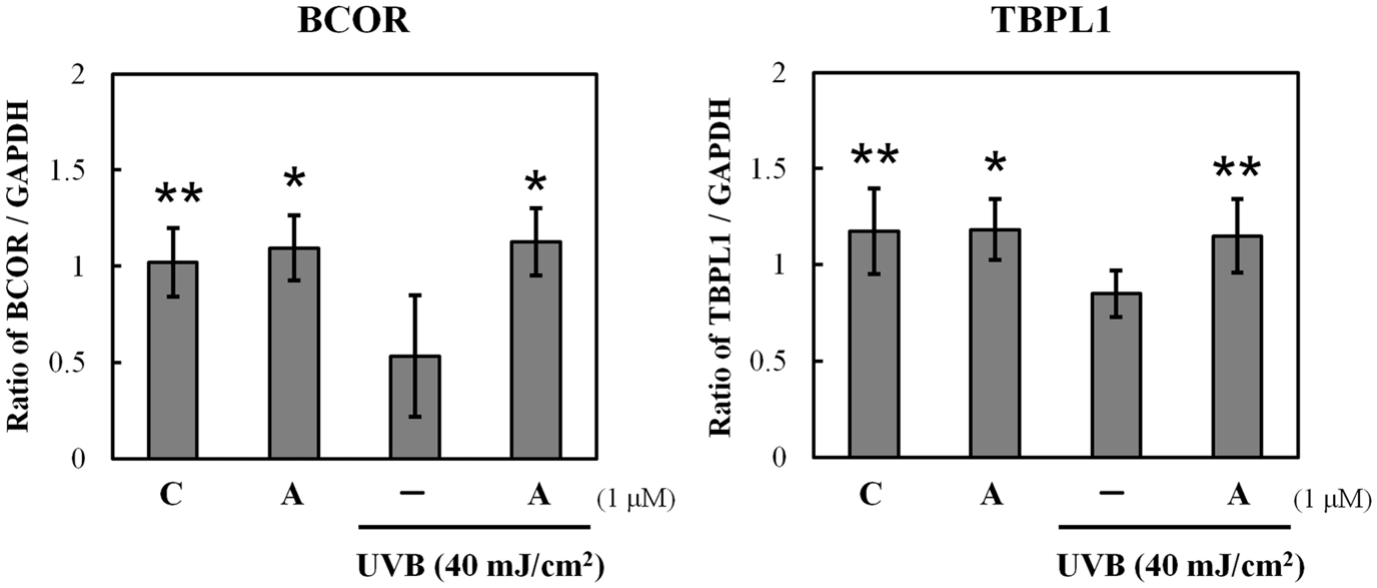
Verification of microarray results by qPCR assay. Cells exposed to alkannin or UVB light (40 mJ/cm^2^) or both were used for real-time qPCR assay of the selected genes BCOR and TBPL1. Each mRNA expression level was normalized with GADPH. Data are presented as mean ± SD (n = 3). *p<0.005, **p<0.05.

### Western Blot Analysis

Cells were collected and washed with cold PBS. Cells were lysed at a density of 1×10^6^ cells/50 µl of RIPA buffer (1 M Tris-HCl, 5 M NaCl, 1% Nonidet P-40 (v/v), 1% sodium deoxycholate, 0.05% SDS, 1 mM phenylmethyl sulfonyl fluoride) for 20 min. Following brief sonication, the lysates were centrifuged at 12,000×*g* for 10 min at 4°C, and the protein content in the supernatant was measured using the Bio-Rad protein assay kit (Bio-Rad, Hercules, CA). Protein lysates were denatured at 96°C for 5 min after mixing with 5 µl SDS-loading buffer applied on an SDS-polyacrylamide gel for electrophoresis, and transferred to nitrocellulose membranes. After incubation with appropriate antibodies, bands were visualized on X-ray film using chemiluminescence ECL detection reagents (Amersham Biosciences, Buckinghamshire, UK) [23]. Band densities were quantified by a BIO-ID image analyzer and the relative amounts of proteins associated with each specific antibody were normalized to the respective β-actin bands.

**Figure 7:**
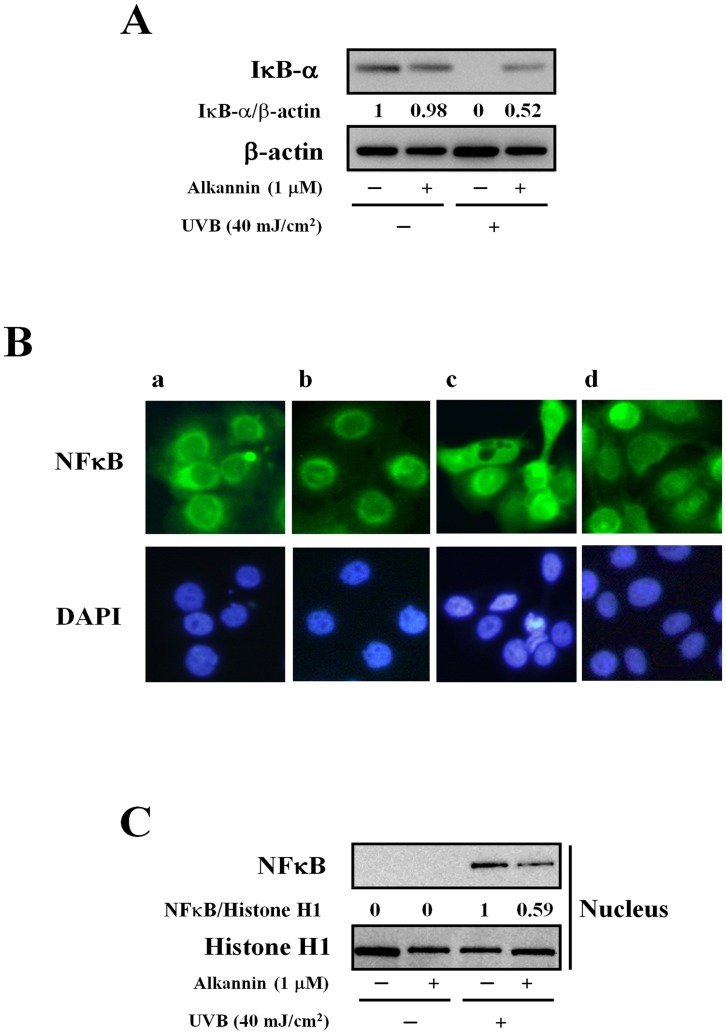
Effects of alkannin on IκB-α activity in UVB-exposed cells. (A) Cells exposed to alkannin or UVB light (40 mJ/cm^2^) alone or both. Western blot analysis was performed with IκB-α protein (30 µg protein in each group). (B) Immunofluorescent assay for the detection of NFκB p65 in: untreated cells (a), cells pre-treated with 1 µM alkannin for 24 h (b), cells exposed to UVB light (c), cells pre-treated with 1 µM alkannin for 24 h and then exposed to 40 mJ/cm^2^ UVB light (d). Results are representative of five independent experiments (original magnification a–d: 200×). (C) NFκB from the nuclear extracts was analyzed by Western blotting, and histone H1 was used as a loading control for nuclear proteins. These data are representative of 3 independent experiments.

### RNA Isolation

Total RNA was extracted from cells using an RNAeasy Total RNA Extraction kit (Qiagen, Valencia, CA) and treated with DNase I (RNase-free DNase kit, Qiagen) for 15 min at room temperature to remove genomic DNA.

### Preparation of Nuclear Extracts

Control and treated cells were washed with ice-cold PBS and centrifuged at 15,000×g for 1 min. Cell pellets were re-suspended in 100 ml of cold lysis buffer (10 mM HEPES/KOH, 0.1 mM EDTA, 10% NP-40, 10 mM KCl, 1 mM DTT, protein inhibitor cocktail, and 0.5 mM phenylmethylsulfonyl fluoride; pH 7.9) by pipetting and incubated on ice for 10 min after. Cells were then centrifuged at 15,000×g for 10 min at 4°C. The nuclear pellets were gently re-suspended in 50 µl of cold extraction buffer (50 mM HEPES/KOH, 300 mM NaCl, 0.1 mM EDTA, 1 mM dithiothreitol, protein inhibitor cocktail, and 0.5 mM phenylmethylsulfonyl fluoride; pH 7.9) for 30 min on ice. After centrifugation (15,000×g for 15 min at 4°C), the supernatant containing nuclear proteins was analyzed.

### GeneChip Analyses

Gene expression was analyzed using a GeneChip system with a Human Genome U133 plus 2.0 Array spotted with about 54,675 probe sets (Affymetrix, Santa Clara, CA, USA). Samples for array hybridization were prepared as described in the Affymetrix GeneChip Expression Technical Manual. Briefly, 500 ng of total RNA was used to synthesize double-stranded cDNAs with a GeneChip 3′ IVT Express kit (Affymetrix). Biotin-labeled cRNAs were then synthesized from the cDNAs using GeneChip Expression 3′-Amplification Reagents for IVT Labeling (Affymetrix). After fragmentation, the biotinylated cRNAs were hybridized to GeneChip array at 45°C for 16 h. The arrays were washed, stained with streptavidin–phycoerythrin, and scanned using a probe array scanner. The scanned chip was analyzed using GeneChip Analysis Suite Software (Affymetrix). The obtained hybridization intensity data were converted into a presence or an absence call for each gene, and changes in gene expression level between experiments were detected by comparison analysis. The data were further analyzed using GeneSpring (Silicon Genetics, Redwood City, CA, USA) to extract the significant genes.

### Real-time Quantitative PCR Assay

Real-time quantitative PCR (qPCR) assay was performed on a real-time PCR system (Mx3000P, Stratagene, Tokyo, Japan) using SYBR PreMix ExTaq (Takara Bio, Shiga, Japan) or Premix ExTaq (for the use of TaqMan probes; Takara Bio) in accordance with the manufacturers’ protocols. Reverse transcriptase reaction (Omniscript Reverse Transcriptase, Qiagen) was carried out with DNase-treated total RNA using an oligo 2 primers; BCOR and TBPL1. Each mRNA expression level was normalized to the mRNA expression level of GAPDH.

### Immunocytochemical Analysis

Cells from different treatments were washed with PBS and fixed with 4% paraformaldehyde (PFA/PBS (v/v); Sigma) for 15 min at 4°C. One% Triton/PBS was added for 5 min to permealize cells. Cells were then incubated with blocking buffer (10% FBS/PBS) for 30 min prior to incubation with the first antibody phospho- NFκB p65 (1∶50 in PBS; Cell Signalling; Celbio, Milan, Italy) for 1.5 h at room temperature. Cells were thoroughly washed and incubated with the secondary antibody conjugated with FITC (1∶200 in 3% FBS/PBS; Sigma). Finally, cells were stained with 1∶100,000 DAPI/PBS (Molecular Probes, Invitrogen) for 2 min for nuclear visualization and thoroughly washed before observation under a fluorescent microscope (Olympus FluoView 500 confocal microscope).

### Statistical Analysis

Differences between various treatments were analyzed using Student’s t-test. For comparisons of multiple groups, one-way ANOVA was applied. P values <0.05 were considered statistically significant. Data in the figures are shown as the mean ± SD.

## Results

### HSP70 Content in HaCaT Cells Exposed to UVB Light

To examine whether the UVB radiation influenced the expression of HSP70, we analyzed the protein production levels in HaCaT cells by western blot at different time intervals, namely, 0, 6, 12, 24, 48, 72, and 96 h, following UVB radiation (40 mJ/cm^2^). As shown in Figure 1, HaCaT cells (control) constitutively expressed HSP70. The relative densities of HSP70 to β-actin were 1.59 and 1.23 in samples irradiated with UVB after 0 and 6 h. On the other hand, cells incubated for 12, 24, 48, 72, and 96 h following irradiation showed decreased HSP70 expression (12 h, 0.35; 24 h, 0.21; 48 h, 0.51; 72 h, 0.73; 96 h, 0.91).

### Effects of HSP70-inducing Agents after UVB Radiation

The effect of HSP70-inducers: geldanamycin, alkannin, oxymatrine, osthole, shikonin, and palmatine chloride, on the production of HSP70 was confirmed by western blot. HaCaT cells were treated with 1 µM of each agent (preliminary investigations at 0.01, 0.1 and 1 µM showed that this concentration range was non-cytotoxic and non-apoptotic for all agents used - Data not shown). The result shows that HSP70 expression level was increased by the treatment with HSP70-inducers at 24 h (Figure 2A). This incensement was sustained for 72 h (Data not shown).

To examine the effect of these agents on HSP70 levels under UVB stress, cells were pretreated with 1 µM of these agents for 24 h before they were exposed to UVB radiation (40 mJ/cm^2^) in the absence of drugs. Cells were then left to incubate for further 24 h in fresh medium before analysis. As shown in Figure 2B, UVB radiation decreased the level of HSP70 in HaCaT cells, whereas the presence of each of the agents increased HSP70 expression to levels higher than that of the control with alkannin showing the highest induction ratio.

The exposure of HaCaT cells to UVB light also resulted in 34.4±1.9% apoptosis detected by DNA fragmentation assay. The percentages of DNA fragmentation in cells exposed to UVB light decreased significantly by the pre-treatment of geldanamycin, alkannin, oxymatrine, osthole, shikonin, and palmatine chloride for 24 h (geldanamycin, 24.1±2.8%; alkannin, 13.6±1.9%; oxymatrine, 15.7±3.1%; osthole, 23.6±3.6%; shikonin, 22.3±3.1%; palmatine chloride, 27.4±3.0%) (Figure 2C). In augmentation, the flow cytometric analysis using annexin V-FITC and PI showed that the percentages of early apoptotic cells decreased from 20.4±1.3% in cells exposed to UVB light to 10.6±1.1–18.1±0.9% in cells pre-treated with HSPs-inducing agents (Figure 2D). The percentages of secondary necrotic cells decreased significantly following UVB radiation only when cells were pre-treated with alkannin.

### Effects of HSP70-inducing Agents on Expression of Caspase-3 Activation after UVB Radiation

Caspases are the important mediators of apoptosis. To determine whether the caspase-dependent mitochondrial pathway is involved in UVB-induced apoptosis and whether it is suppressed by the pre-treatment with the HSP70-inducing agents used in the present study, we measured the intracellular caspase-3 activities at 24 h after UVB radiation in the presence or absence of each agent. Figure 3 shows that caspase-3 activity was detected post UVB treatment, while it was significantly decreased in the presence of geldanamycin, alkannin, oxymatrine, osthole, and shikonin with alkannin displaying the strongest suppressant effect.

### Inhibitory Effects of a HSP70 Inhibitor KNK437 on UVB-induced Apoptosis

To confirm that the alkannin-induced expression of HSP70 was related to the suppression of UVB-induced apoptosis, we examined the effect of the HSP70 inhibitor (KNK437) on HSP70 levels and UVB-induced apoptosis. KNK437 addition resulted in a significant dose-dependent decrease in HSP70 expression (Figure 4A), which was accompanied by a corresponding increase in UVB-induced apoptosis (Figure 4B).

### GeneChip Analysis

Most studies on heat shock stress examine the induction of HSP70. However, there are several families of HSPs each with different molecular chaperone functions. Here, we carried out GeneChip analysis to compare between gene expression in UVB radiation (40 mJ/cm^2^) group and in UVB radiation and alkannin combination group, using RNAs isolated from respective cells at 24 h after UVB radiation. Genes of our interest with mean fold change greater than 3.00 or less than 0.33 were listed in Table 1. The peak gene expression of HSPs, such as HSPA13 and HSPA5, was observed following UVB exposure in cells pre-treated with alkannin. Moreover, neither DNA damage-inducible SOD2 nor SERPINE1 nor GADD45B gene expression was up-regulated in spite of HSP protein accumulation following UVB exposure. Furthermore, to determine the biologically relevant network and pathway of the genes related to HSPs, pathway analysis of the down or up-regulated genes was carried out using Ingenuity Pathways Analysis Knowledge Base (Figure 5). The expression level of the 4 genes DNAJB9, DNAJB12, BCOR and TBPL1 were down-regulated by UVB radiation and then up-regulated by the combination (Figure5A, B).

To confirm the results of gene chip analysis, a real-time qPCR assay was performed for two selected genes in the network, namely, BCOR and TBPL1. As observed with gene chip results, the expression level of these genes was significantly increased by the pre-treatment of cells with alkannin (Fig. 6).

### Effects of HSP70-inducing Agent on NFκB Activation Pathway after UVB Radiation

HSP70 was reported to suppress the activation of NFκB through various pathways including the suppression of the inflammatory stimuli-induced degradation of NFκB inhibitor (IκB-α) [24]. To determine whether this pathway occurs with alkannin pre-treatment, we studied the effect of alkannin on IκB-α production level. As shown in Figure 7A, UVB radiation decreased the level of IκB-α, whereas the level remained significantly high in alkannin pre-treated cells. In support to the alteration of IκB-α levels, the expression levels of some pro-inflammatory cytokine genes regulated by NFκB, such as IL-1β, IL-6 and IL-8, were down-regulated in the combination group (Data not shown). These results suggest that expression of HSP70 in HaCaT cells suppresses the UVB-induced IκB-α degradation which in turn suppresses NFκB activity as shown in Figure 7B and C.

### Effect of Alkannin on NFκB Nuclear Translocation

In parallel, the nuclear fraction of the cells was prepared and analyzed by Western blotting. As shown in Figure 7C, UVB-induced nuclear translocation of NFκB was suppressed by alkannin pretreatment.

## Discussion

UV radiation of the skin results in a variety of injuries involving inflammatory and repair reactions, free radical reactions, and apoptosis. In particular, UVB radiation, which is known to damage epidermal cells, contributes to several pathological conditions that include epidermal photo-aging, photo-damage and photo-carcinogenesis, immunosuppression, inflammation (activation of pro-inflammatory cytokines and chemokines), and DNA damage [25], [26]. In the skin, a delicate balance should be maintained between keratinocyte proliferation and cell death to ensure terminal differentiation and cornification in an orderly manner that is coordinated throughout all layers of the human epidermis [27]. When this balance is disturbed by UVB radiation, the cells cannot repair the resulting DNA damage, resulting in apoptotic sunburn cells formation [28]. Therefore, the suppression of UVB-induced damage (apoptosis) in keratinocytes is beneficial for the prevention of photo-damage. In the present study, we demonstrate a novel mechanism by which alkannin could prevent UVB-induced apoptosis. Our data show that alkannin, as well as oxymatrine, osthole, palmatine chloride and shikonin, could effectively reduce cell death and apoptotic DNA cleavage after UVB radiation through the induction of HSPs.

The most studied family of anti-apoptotic proteins is the HSP70 family proteins which consists of both constitutively expressed and inducible (mainly HSP72) members. The induction of HSP70 has been reported after exposure to arsenic, heavy metals, infrared laser radiation and heat shock [29]–[32]. However, research related to the effects of UV radiation on the expression of these proteins is scarce [33]–[35]. *In vitro* studies showed that the artificial expression of HSP70 in keratinocytes confers protection against UVB light [36]–[39]. The protective role of HSP70 against UVB-induced epidermal damage was also suggested by *in vivo* studies in which the whole body hyperthermia of mice prevented UVB-induced sunburn cell formation, whereas HSP70-null mice showed a sensitive phenotype to UVB-induced epidermal damage [40]–[42]. HSP70-mediated protection of the skin against UVB light has been also reported to occur in human skin [41]. We also found that pretreatment of cells with alkannin caused significant decrease in UVB-induced cleavage of caspase-3, and the addition of KNK437 reversed the action of alkannin increasing UVB-induced apoptosis in a dose-dependent manner. HSPs have been shown to block apoptosis by interfering with caspase activation [43], [44]. With the high-density oligonucleotide microarrays and computational gene expression tools, we identified a unique gene network containing HSPs genes, such as HSPA, BCOR and DNAJB. HSPA and DNAJ proteins function as molecular chaperones to assist in processes such as translation and transport of proteins across membranes. The BCOR plays an important role in the function and survival of certain immune system cells. In the present study, after alkannin pre-treatment BCOR and DNAJB up-regulation was observed. It is well known that the pro-inflammatory cytokine pathways, such as TNF-α are also involved in apoptosis evoked by UV radiation. HSP70 was reported to suppress the activation of NFκB through various pathways such as the suppression of the inflammatory stimuli-induced degradation of NFκB inhibitor (IκB-α) [24]. In support to the previous reports, microarray data also identified that the UVB-induced pro-inflammatory cytokines, such as IL-1β, IL-6 and IL-8, were abrogated in cells pre-treated with the HSP70-inducing drug alkannin (Data not shown).

In conclusion, our results indicate that alkannin inhibits the UVB-induced apoptosis in HaCaT cells. Although the detailed mechanisms by which alkannin induces the expression of HSP70 are not fully understood, it is possible that HSPA13, HSPA5 and their associated genes could participate in the up-regulation of HSP70. Consequently, alkannin pre-treatment appears to be beneficial in the photoprotection of the skin. The elucidation of the molecular mechanisms of HSP70 induction by alkannin remains for further investigation in the future.
